# Editorial: HIV-1 Tat, an enhancer of virus infectivity and disease promoter: target for preventive and therapeutic interventions

**DOI:** 10.3389/fimmu.2026.1881370

**Published:** 2026-06-26

**Authors:** Aurelio Cafaro, Jonathan Karn, Ben Berkhout, Susana Valente, Barbara Ensoli

**Affiliations:** 1 National HIV/AIDS Research Center, Istituto Superiore di Sanità, Rome, Italy; 2 Department of Molecular Biology and Microbiology, School of Medicine, Case Western Reserve University, Cleveland, OH, United States; 3 Laboratory of Experimental Virology, University of Amsterdam, Amsterdam, Netherlands; 4 Department of Immunology and Microbiology, University of Florida, Scripps Biomedical Research, Jupiter, FL, United States

**Keywords:** AIDS- and non-AIDS-associated morbidities, anti-Tat antibodies, HIV cure, HIV reservoir, HIV-1 Tat, preventive and therapeutic interventions, transmission

Although antiretroviral therapy (ART) effectively suppresses plasma viremia, individuals with HIV still experience ongoing immune activation, tissue dysfunction, and non-AIDS-related health issues. This highlights the biological impact of residual viral activity that persists despite treatment. Among HIV-1 regulatory and accessory proteins, Tat (Trans-Activator of Transcription) occupies a distinctive position, coordinating viral transcription with both intracellular signaling and extracellular effects. It is produced early after infection and is necessary for efficient HIV gene expression (1). Tat is also actively released from infected cells and remains detectable during ART, indicating persistent low-level HIV transcription that current therapies do not fully suppress (2–4). As both an intracellular regulator and an extracellular effector, Tat modulates diverse cellular pathways, linking residual viral transcription to immune dysregulation, tissue injury, and reservoir maintenance. Moreover, immune responses against Tat—particularly anti-Tat antibodies—are linked to slower disease progression and better ART responses (5). Because of these diverse activities, Tat occupies a strategic position in emerging HIV cure paradigms, particularly in therapies targeting latency reversal (“shock-and-kill”) and transcriptional silencing (“block-and-lock”).

This Research Topic features studies on Tat’s roles in HIV-1 infection and disease, as well as the potential for Tat-targeted therapies. For clarity, intracellular and extracellular activities of Tat are discussed separately, although they are mechanistically interconnected. Because Tat is efficiently taken up by neighboring cells (6, 7), it can modulate gene expression and viral transcription in both infected and uninfected cells.

Recent research emphasizes the role of intracellular Tat in causing lasting changes in host cell biology. Andrea Rodrìguez-Agustìn et al. found persistent DNA methylation changes in Tat-expressing Jurkat cells, particularly in regions regulating genes involved in survival, inflammation, antigen presentation, and immune responses. Similarly, Casanova et al. showed that Tat expression in Jurkat cells and primary CD4+ T cells increases aging markers and encourages the secretion of senescence-related factors. These findings support a model in which Tat induces epigenetic reprogramming and early cellular aging, which may lead to ongoing inflammation and immune imbalance in people with HIV (PWH), even when the virus is virologically suppressed (8).

These effects extend to tissue-specific compartments, including the central nervous system (CNS), where limited drug penetration and ongoing transcription sustain viral protein production. HIV-associated neurocognitive disorders (HAND) remain prevalent among ART-treated PWH, though estimates vary by diagnostic criteria. Using *in vitro* and *in vivo* models, Periyasami et al. identify a mechanism by which Tat induces CCL2 (MCP-1) expression in microglial cells, thereby promoting neuroinflammation. This pathway links persistent viral transcription to CNS immune activation and may represent a therapeutic target.

A defining feature of Tat biology is its extracellular activity. During active transcription, Tat is released and binds to extracellular matrix components, including heparan sulfate proteoglycans (9), creating gradients that recruit target cells via chemokine mimicry and facilitate viral spread (10). Beyond this role in transmission, extracellular Tat alters the phenotype and function of both susceptible and non-susceptible cells. This capacity to modulate uninfected cells provides a plausible mechanism for the systemic manifestations of HIV disease, including the increased incidence and aggressiveness of malignancies such as Kaposi’s sarcoma (11, 12).

Tugizov reviews the effects of extracellular Tat on epithelial and endothelial cells, demonstrating activation of the MAPK, NF-κB, and TGF-β signaling pathways. These pathways disrupt tight and adherens junctions, compromise barrier integrity, and promote epithelial–mesenchymal transition, a phenotype associated with increased invasiveness and tumorigenesis (13). Complementing these findings, Chandran et al. describe how Tat induces endothelial dysfunction through oxidative stress, inflammatory signaling, mitochondrial impairment, and increased cellular adhesion. These processes likely contribute to vascular pathologies affecting multiple organ systems, including the brain, heart, and lungs, and provide a mechanistic basis for non-AIDS comorbidities observed in PWH.

The dual role of Tat in pathogenesis and therapeutic targeting is evident in studies of latency reversal. Fisher et al. review Tat-based latency-reversing agents (LRAs), including nanoparticle delivery strategies to improve targeting. These approaches highlight a central challenge: while Tat contributes to HIV persistence and pathology, its potent transactivation function also makes it an attractive candidate for latency reversal. However, delivery, specificity, and safety issues remain significant barriers to clinical translation, underscoring the need for approaches that balance efficacy with risk.

The pathogenic effects of Tat can be counteracted by immune responses. Anti-Tat antibodies, although infrequent in PWH, are associated with delayed disease progression and improved immunologic outcomes (14–16). Preclinical studies in nonhuman primates have shown that Tat vaccination can prevent or contain infection with pathogenic SHIV strains (17, 18). Clinical studies further suggest that Tat immunization in ART-treated individuals enhances immune restoration and accelerates proviral DNA decay (19–22). In this Research Topic, Picconi et al. report long-term follow-up of a randomized phase II trial in South Africa, demonstrating sustained improvements in CD4+ T-cell counts and continued decline in proviral load over 12 years without booster immunization. These findings support continued evaluation of Tat-based immunization strategies, although confirmation in larger and more diverse populations will be required. The potential to mitigate the effects of suboptimal ART adherence further supports their relevance in real-world and resource-limited settings.

In summary, the studies in this Research Topic portray Tat as more than a transcriptional regulator and identify it as a key mediator linking ongoing HIV transcription to immune activation, tissue dysfunction, and reservoir persistence. Tat’s dual role at the cellular and extracellular levels provides a critical link between viral survival and host pathology. Targeting Tat or its regulated pathways could offer opportunities to address various aspects of HIV, including residual inflammation, comorbidities, and the stability of the viral reservoir. As cure strategies increasingly incorporate latency reversal, immune modulation, and transcriptional regulation, Tat emerges as a central target that can be leveraged across each of these approaches. A deeper understanding of Tat biology is essential for developing interventions aimed not only at maintaining viral suppression but also at achieving long-term control of HIV-related disease.
